# Effect of traffic pollution on respiratory and allergic disease in adults: cross-sectional and longitudinal analyses

**DOI:** 10.1186/1471-2466-9-42

**Published:** 2009-08-24

**Authors:** Mar Pujades-Rodríguez, Tricia McKeever, Sarah Lewis, Duncan Whyatt, John Britton, Andrea Venn

**Affiliations:** 1 Division of Epidemiology and Public Health, University of Nottingham, Nottingham, UK; 2 Department of Geography, University of Lancaster, Lancaster, UK

## Abstract

**Background:**

Epidemiological research into the role of traffic pollution on chronic respiratory and allergic disease has focused primarily on children. Studies in adults, in particular those based on objective outcomes such as bronchial hyperresponsiveness, skin sensitisation, and lung function, are limited.

**Methods:**

We have used an existing cohort of 2644 adults aged 18–70 living in Nottingham, UK, for whom baseline health and demographic data were collected in 1991 and computed two markers of exposure to traffic: distance between the home and nearest main road and modelled outdoor nitrogen dioxide (NO_2_) concentration at the home location. Using multiple regression techniques, we analysed cross-sectional associations with bronchial hyperresponsiveness, FEV_1_, spirometry-defined COPD, skin test positivity, total IgE and questionnaire-reported wheeze, asthma, eczema and hayfever in 2599 subjects, and longitudinal associations with decline in FEV_1 _in 1329 subjects followed-up nine years later in 2000.

**Results:**

There were no significant cross-sectional associations between home proximity to the roadside or NO_2 _level on any of the outcomes studied (adjusted OR of bronchial hyperresponsiveness in relation to living ≤150 m vs >150 m from a road = 0.92, 95% CI 0.68 to 1.24). Furthermore, neither exposure was associated with a significantly greater decline in FEV_1 _over time (adjusted mean difference in ΔFEV_1 _for living ≤150 m vs >150 m of a road = 10.03 ml, 95% CI, -33.98 to 54.04).

**Conclusion:**

This study found no evidence to suggest that living in close proximity to traffic is a major determinant of asthma, allergic disease or COPD in adults.

## Background

Many epidemiological studies have examined effects of exposure to road vehicle traffic on chronic respiratory and allergic disease in children, but research of the effects in adults is limited. A handful of studies of adults have reported that living in close proximity to busy or major roads is associated with an increased risk of wheeze [1-3], whilst others have shown no effect on wheeze or asthma [4-9]. Some of this inconsistency may be due to the use of self-reported markers of asthma which are potentially biased, but use of objective markers such as bronchial hyperresponsiveness (BHR) is rare. Lung function measures such as one second forced expiratory volume (FEV_1_) have been investigated by a few in relation to traffic indices such as proximity to main roads or modelled traffic-related pollutants, but again findings in adults are inconclusive and evidence of longitudinal effects lacking[10]. Spirometry has also been used to define chronic obstructive pulmonary disease (COPD) in one study of women which reported an adverse effect of close residential proximity to a busy road[11]. Investigations of allergy and atopy in adults have also tended to rely on self-reported outcomes, and use of objective markers such as skin sensitisation or elevated immunoglobulin E (IgE) rare[6,8].

We have therefore used data from an existing population-based cohort of adults to compute markers of exposure to traffic-related pollution and investigate their relation with objective measures of respiratory and allergic disease, namely bronchial hyperresponsiveness, FEV_1_, spirometry-defined COPD, skin test positivity and total IgE, as well as questionnaire reported wheeze, asthma, eczema and hayfever. In addition to these cross-sectional investigations, we have also used longitudinal measurements made on the cohort to examine effects of exposure on change in FEV_1_. As the primary traffic related air pollutants are highest at the roadside and decline exponentially[12], we have chosen to use distance between home residence and the nearest major road as an objective proxy of exposure to traffic pollution, and to look particularly at dose-response relations across the first 150 m from the roadside where most of the decline occurs. We have also used an alternative marker of exposure based on modelled traffic-related NO_2 _at the home location, which may better reflect the level and type of traffic. Data on number of years residence in the current home has also enabled us to look more specifically at long-term effects of exposure by restricting analyses to long-term residents only.

## Methods

### Study population

**Figure 1:**
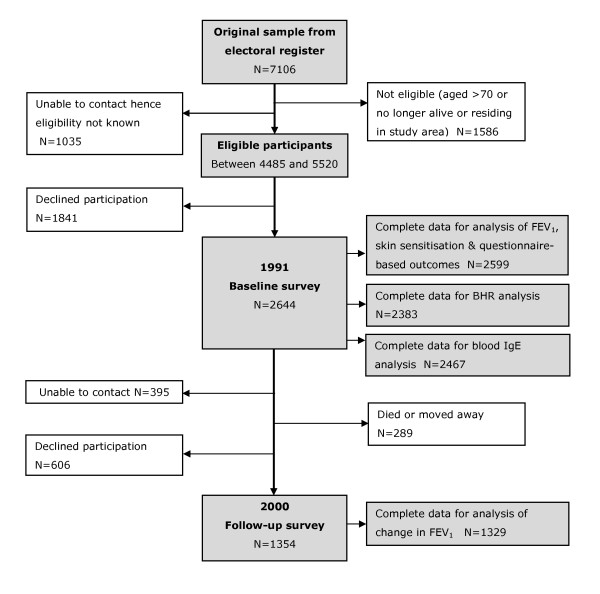
**Flow diagram showing study participation**.

Our study population is a cohort of adults aged 18–70 living in the Gedling area of Nottingham City, UK, who were recruited in 1991 as part of a study of the effect of diet on chronic lung disease. Gedling is an area of 46 square miles with an estimated population of 87,000 in 1991 which covers the north east suburbs of Nottingham and surrounding rural villages. Full details of the study have been described elsewhere[13]. Briefly, a representative sample of adults was drawn from the local electoral register and those of eligible age and residing in the study area were invited to take part in the study (figure 1). Information on respiratory and allergic disease symptoms, demographics, smoking, diet, and numerous other lifestyle factors were collected using an interview-led questionnaire. FEV_1 _and forced vital capacity (FVC) were measured by a study nurse using a calibrated dry bellows spirometer (Vitalograph, Buckingham, UK) taking the best of three technically satisfactory manoeuvres with the subject seated; a methacholine challenge performed to determine BHR using the technique described by Yan et al[14]; allergen skin tests to *Dermatophagoides pteronyssinus*, mixed grass pollen, cat fur, *Aspergillus fumigatus*, and *Cladosporium herbarum *(Bencard solutions, Brentford, UK) carried out; and a blood sample taken. In total, 2,644 individuals participated in the 1991 survey, estimated to be between 48% and 59% of those eligible [13] (figure 1). The exact response rate cannot be computed since it was not known what proportion of the non-responders would have been eligible for inclusion. In 2000 all surviving individuals were invited to participate in a follow-up survey (follow-up rate was 51%) in which these measurements, with the exception of BHR, were repeated[15]. The surveys were approved by the Nottingham City Hospital and Nottingham University ethics committees.

### Computation of exposure variables

We took each subjects home address in 1991 and converted it into a grid reference using information from the Local Land & Property Gazetteers database held by Gedling Borough Council. To compute our distance exposure variable, we linked this grid reference to a digitised road map of Great Britain (Meridian database; Ordnance Survey, Southampton, UK), which is a geometrically structured 1:50,000 scale vector database with a coordinate resolution of 1 m. We then calculated the shortest distance (in metres) between each address location and the nearest major road, defined as a motorway (freeway), or 'A' or 'B' class road (principal road as classified by UK Department for Transport), using Geographical Information System (GIS) software (ArcGIS 9.0).

To compute our modelled NO_2 _variable, we linked each home location grid reference to a high resolution map of modelled traffic-related NO_2 _using ArcGIS. This map was generated by the dispersion model ADMS Roads (CERC, Cambridge, UK), which has been widely used to assess the impact of road traffic on local air quality and extensively validated against monitored roadside concentrations of traffic pollutants[16]. Traffic count and composition data supplied by Nottinghamshire County Council and hourly sequential meteorological data (including wind direction and speed) provided by the UK Meteorological Office were inputted into the model. Background concentration data were averaged from pollutant data recorded at automated sites in Nottingham City Centre (urban) and Sutton Bonnington (rural). Modelled annual mean concentrations of NO_2 _were verified through comparison with observed concentrations (1998–2003) recorded at 10 diffusion tube survey sites (background, intermediate and roadside) across the study area and overall modelled values correlated well with observed (r = 0.63). Closer inspection revealed some underestimation of observed concentrations at roadside sites close to major road intersections where slow moving or standing traffic is likely during rush hours. In the absence of more detailed information on traffic count, speed and composition the model is unable to reproduce elevated concentrations at such localized hot spots. However given the good level of agreement between modelled and observed concentrations elsewhere (r = 0.88 for the other sites), and the fact that only a minority of the study population would live close to such major intersections, the model parameterisation was deemed satisfactory to invoke deployment across the entire study area at a spatial resolution of 10 metres.

### Statistical analyses

In cross-sectional analyses we assessed the effect of each exposure variable on each of the following outcome variables using data from the 1991 baseline survey: self-reported wheeze in the past year ('Have you had wheezing or whistling in your chest at any time in the last 12 months?'), diagnosed asthma ('Have you ever had asthma?' and 'Was this confirmed by a doctor?'), eczema ever ('Have you ever had eczema or any kind of skin rash?') and hayfever ever ('Have you ever had hayfever or other nasal allergies?'); bronchial hyperresponsiveness (BHR), defined as a methacholine dose provoking a 20% fall in FEV_1 _(PD_20_) of 12.25 mg/ml or less; COPD, defined as stage I or above using the GOLD (Global Initiative for Chronic Obstructive Lung Disease) criteria[17] (namely having an FEV_1_/FVC less than 70%); allergen skin sensitisation, defined as a response to any of the allergens tested at least 3 mm greater than the saline control response in the presence of a positive histamine control; and high total IgE, defined as a concentration above 100 kU/l. Multiple logistic regression analyses were carried out to assess the effect of distance, initially treated as a binary variable (<= 150 m and >150 m), on each binary outcome, adjusted for age, sex, smoking status (current, never or ex-smoker) and quintiles of Carstairs deprivation score (postcode based index of deprivation based on unemployment, overcrowding, car ownership and occupation[18]). To investigate dose-response effects, analyses were then carried out in the subset of respondents living within 150 m from a road with distance fitted as 50 m bands (the smallest categorisation possible given the study sample size). Modelled NO_2 _was categorised into quintiles and analysed in a similar way to distance.

The cross-sectional association between each exposure and FEV_1_, defined as the best of three satisfactory measurements, was analysed using multiple linear regression controlling for sex, age, age squared, smoking status, pack-years of cigarettes smoked, height, Carstairs deprivation score and age-height interaction. Multiple linear regression was also used to analyse total IgE as a continuous variable (transformed to achieve a Normal distribution by adding 1 and taking the logarithm), with adjustment for age, sex, smoking and Carstairs score.

In longitudinal analyses, we computed changes in adjusted FEV_1 _residuals between 1991 and 2000 using the method described by Carey *et al*[19]. Briefly, we modelled predicted FEV_1 _values for each sex separately in the sub-group of non-smoking, non-asthmatic, non-wheezing individuals, with terms for age, height, age squared, and age-height interaction, and calculated the difference from predicted as the adjusted FEV_1 _residuals in 1991 and 2000. Change in FEV_1 _residual from 1991 to 2000 was modelled in relation to the exposure variables using multiple linear regression, controlling for the effect of smoking and number of pack-years of cigarettes in 2000 and for Carstairs deprivation score as recorded at baseline.

For all analyses we tried adjusting for other potential confounders including exposure to passive smoking, older siblings, indoor heating and cooking appliances, pet ownership, vitamin C and E intake, and body mass index. We also tried controlling for social class based on occupation as an alternative marker of socio-economic status to Carstairs score. Since long-term exposure may be most relevant and inclusion of others could weaken any associations, we repeated all cross-sectional analyses restricting to the subgroup of individuals who had lived in the same address for at least 3 years (long-term residents), and longitudinal analysis restricting to individuals who lived in the same address between 1991 and 2000, to assess whether the magnitude of effect estimates were altered.

All analyses were performed in STATA 8.2 for Windows (Stata Corportation, college Station, Texas). The available sample provided 80% power to detect an odds ratio (OR) of 1.35 for wheeze (based on an outcome prevalence of 24%) in relation to living within 150 m of a road relative to more than 150 m (based on an exposure prevalence of 23%).

## Results

### Population

Of the 2,644 participants in the 1991 baseline survey, we excluded 44 with invalid or incomplete address information and one who had not provided full lung function data. Cross-sectional analyses were therefore carried out on 2,599 subjects (98%), of whom 2,351 (90.5%) were long-term residents (median length of residence 10 years; interquartile range 5 to 20 years). For the longitudinal analyses we excluded 14 without valid address information and 11 without lung function data, leaving 1,329 individuals (98.2% of participants in the follow-up survey) for analysis, of whom 997 (75.0%) had resided at the same address over the period of follow-up. The baseline characteristics of the study subjects and those lost to follow-up are shown in Table 1. Those followed up in 2000 were generally similar to the 1991 baseline group, although slightly less likely to be a smoker or in the youngest age group. Although the characteristics of our original 7106 adults sampled from the electoral role are not known, Table 2 shows how the age, sex and social deprivation (Carstairs) distribution of our participants compares with that of all Gedling residents in 1991, our target population, using census data from that year [20] (Table 2). This shows that those included in our cross-sectional and longitudinal analyses were slightly older than the target population but similar with respect to gender and social deprivation (Table 2). Cross-sectional analyses of BHR and IgE were carried out on slightly smaller datasets due to missing data (figure 1), but demographics were similar to the complete dataset of 2599 subjects (Table 2).

**Table 1:** Baseline characteristics of Gedling study participants included in cross-sectional and longitudinal traffic pollution analyses and those lost to follow-up

**Baseline characteristics**	**Cross-sectional analysis** **(N = 2599) Number (%)**	**Longitudinal analysis** **(N = 1329) Number (%)**	**Lost to follow-up** **(N = 1267) Number (%)**
**Age group**			
18–29	415 (16.0)	134 (10.1)	281 (22.2)
30–39	553 (21.3)	263 (19.8)	290 (22.9)
40–49	664 (25.6)	369 (27.8)	295 (23.3)
50–59	523 (20.1)	341 (25.7)	181 (14.3)
60–70	444 (17.1)	222 (16.7)	220 (17.4)
**Sex**			
Men	1299 (50.0)	659 (49.6)	639 (50.4)
Women	1300 (50.0)	670 (50.4)	628 (49.6)
**Carstairs deprivation quintiles**			
1^st ^(least deprived)	636 (24.5)	292 (22.0)	282 (22.3)
2^nd^	457 (17.6)	255 (19.2)	202 (15.9)
3^rd^	523 (20.1)	307 (23.1)	241 (19.0)
4^th^	482 (18.6)	220 (16.6)	264 (20.8)
5^th ^(most deprived)	501 (19.3)	255 (19.2)	278 (21.9)
**Smoking status**			
Never	1289 (49.6)	705 (53.1)	583 (46.0)
Ex-smoker	722 (27.8)	387 (29.1)	334 (26.4)
Current smoker	588 (22.6)	237 (17.8)	350 (27.6)
**Distance to a main road**			
<50 m	164 (6.3)	87 (6.6)	77 (6.1)
50 – 100 m	226 (8.7)	112 (8.4)	114 (9.0)
100 – 150 m	190 (7.3)	96 (7.2)	94 (7.4)
> 150 m	2019 (77.7)	1034 (77.8)	982 (77.5)
**Modelled quintiles of NO_2 _(μg/m^3^)**			
<33.92	520 (20.0)	267 (20.1)	252 (19.9)
33.92 – 34.23	520 (20.0)	279 (21.0)	240 (18.9)
34.23 – 34.73	520 (20.0)	277 (20.8)	242 (19.1)
34.73 – 36.79	520 (20.0)	248 (18.7)	272 (21.5)
>36.79	519 (20.0)	258 (19.4)	261 (20.6)
Outcomes*			
Current wheeze	624 (24.0)	274 (20.6)	347 (27.4)
Diagnosed asthma	231 (8.9)	102 (7.7)	128 (10.1)
COPD**	238 (9.2)	102 (7.7)	135 (10.7)
BHR^†^	310 (13.0)	147 (11.7)	163 (12.9)
Ever hay fever	662 (25.5)	323 (24.3)	337 (26.6)
Ever eczema	714 (27.5)	365 (27.5)	348 (27.5)
Allergen skin sensitisation	788 (30.3)	406 (30.6)	381 (30.1)
High total IgE^‡^	549 (22.3)	258 (20.2)	290 (22.9)

NO_2 _nitrogen dioxide; COPD chronic obstructive pulmonary disease; BHR bronchial hyper-responsiveness; IgE immunoglobulin E.

* For outcomes, values represent number and % positive where %'s are computed excluding any missing values

**COPD: 7 missing values in cross-sectional study (% = 238/2592) and 1 in longitudinal (% = 102/1328)

^† ^BHR: 216 missing values in cross-sectional study (% = 310/2383) and 74 in longitudinal (% = 147/1255)

^‡ ^IgE: 132 missing values in cross-sectional study (% = 549/2467) and 53 in longitudinal (% = 258/1276)

**Table 2:** Comparison of Gedling study participants included in data analyses with 1991 Gedling census population

**Characteristic**	**1991 Gedling** ** census pop^n^** [20]*	**Main cross-** **sectional analyses ** **N = 2599**	**BHR ** **analysis** **N = 2383**	**IgE ** **analysis** **N = 2467**	**Longitudinal** ** analysis of FEV_1_** **N = 1329**
**Age group (%)**					
18–29	24.9	16.0	16.5	15.2	10.1
30–39	21.2	21.3	21.7	21.2	19.8
40–49	22.0	25.6	27.1	26.1	27.8
50–59	16.7	20.1	20.3	20.2	25.7
60–70	15.2	17.1	14.5	17.2	16.7
					
**Sex (%)**					
Men	49.4	50.0	50.4	50.1	49.6
Women	50.6	50.0	49.6	49.9	50.4
					
**Mean (SD) Carstairs deprivation score**	-1.29 (2.05)	-1.32 (2.17)	-1.34 (2.16)	-1.32 (2.17)	-1.53 (2.15)

BHR bronchial hyper-responsiveness; IgE immunoglobulin E.

*Age and sex %'s based on Gedling 1991 census population aged 18–69 (n = 74899); mean Carstairs based on total Gedling 1991 census population (n = 110127)

Census output is Crown copyright and is reproduced with the permission of the Controller of HMSO and the Queen's Printer for Scotland.

Just under one quarter of individuals (22.3%, n = 580) lived within 150 m of a main road and the median level of modelled NO_2 _exposure was 34.39 μg/m^3 ^(IQR 34.01 to 35.94). The concentration of modelled NO_2 _was significantly related to residential proximity to roads (chi square p-value < 0.0001) such that those living within 30 m of a road had a median level of 39.79 μg/m^3 ^(IQR 38.13 to 42.34) decreasing to 34.22 μg/m^3 ^(IQR 33.92 to 34.71) for those living more than 150 m away.

### Effect of proximity to a major road on respiratory and allergic outcomes

After adjusting for potential risk factors, respondents living within 150 m of a major road were not more likely to have BHR, COPD, positive skin test or high total IgE, or self-reported wheeze, than those living further away (Table 3). For wheeze and allergen sensitisation there was weak evidence of a positive dose-response relation across the first 150 m from the roadside (p for trend = 0.07 and 0.03 respectively), but not for the other outcomes. There were no significant associations between proximity and questionnaire-reported asthma, eczema and hay fever (adjusted OR (95% CI) 0.95 (0.68 to 1.33), 0.98 (0.78 to 1.22) and 1.00 (0.81 to 1.25) respectively).

**Table 3:** Association between residential proximity to a main road and respiratory and allergic outcomes

**Outcomes**	**Number with outcome (%)**	Adjusted OR*(95% CI)	**p-value^†^**
**Wheezing in last year**			
≤150 m	129 (22.2)	0.86 (0.68 – 1.08)	0.19
>150 m	495 (24.5)	1	
*Bands of distance*			
<50 m	46 (28.1)	1.60 (0.96 – 2.68)	0.07
50 – 100 m	45 (19.9)	1.00 (0.61 – 1.66)	
100 – 150 m	38 (20.0)	1	
**COPD**			
≤150 m	53 (9.2)	0.97 (0.68 – 1.37)	0.86
>150 m	185 (9.2)	1	
*Bands of distance*			
<50 m	16 (9.8)	1.54 (0.69 – 3.45)	0.29
50 – 100 m	24 (10.7)	1.67 (0.79 – 3.49)	
100 – 150 m	13 (6.8)	1	
**BHR**			
≤150 m	64 (11.9)	0.92 (0.68 – 1.24)	0.57
>150 m	246 (13.3)	1	
*Bands of distance*			
<50 m	13 (8.5)	0.54 (0.27 – 1.11)	0.09
50 – 100 m	25 (12.0)	0.80 (0.44 – 1.45)	
100 – 150 m	26 (14.7)	1	
**Allergic sensitisation**			
≤150 m	163 (28.1)	0.87 (0.70 – 1.07)	0.19
>150 m	625 (31.0)	1	
*Bands of distance*			
<50 m	51 (31.1)	1.72 (1.05 – 2.81)	0.03
50 – 100 m	70 (31.0)	1.53 (0.97 – 2.42)	
100 – 150 m	42 (22.1)	1	
**High total IgE**			
≤150 m	109 (19.7)	0.80 (0.63 – 1.02)	0.07
>150 m	440 (23.0)	1	
*Bands of distance*			
<50 m	31 (20.3)	1.21 (0.69 – 2.12)	0.51
50 – 100 m	43 (19.6)	1.01 (0.61 – 1.69)	
100 – 150 m	35 (19.3)	1	

OR odds ratio; CI confidence interval; COPD chronic obstructive pulmonary disease; BHR bronchial hyper-responsiveness; IgE immunoglobulin E.

* OR adjusted for sex, age group, Carstairs deprivation scores and smoking status

† p-value for trend across bands of distance

### Effect of modelled NO_2 _on respiratory and allergic outcomes

There was no evidence that increasing modelled NO_2 _at the home was related to an increase in the risk of wheeze, COPD, BHR, skin sensitisation or high IgE (Table 4). Similarly, questionnaire reported asthma, eczema and hayfever were not significantly related to modelled NO_2 _(adjusted OR for highest versus lowest quintile 0.96 (0.62 to 1.49), 1.07 (0.81 to 1.41), and 1.02 (0.77 to 1.37), respectively).

**Table 4:** Association between modelled NO_2 _level and respiratory and allergic outcomes

**Quintiles of modelled NO_2 _(μg/m^3^)**	**Number with outcome (%)**	Adjusted OR* (95% CI)	**p for trend**
**Wheezing in last year**			
<33.92	124 (23.9)	1	0.18
33.92 – 34.23	134 (25.8)	1.03 (0.76 – 1.39)	
34.23 – 34.73	122 (23.5)	0.86 (0.63 – 1.16)	
34.73 – 36.79	122 (23.5)	0.84 (0.63 – 1.14)	
>36.79	122 (23.5)	0.88 (0.66 – 1.19)	
**COPD**			
<33.92	46 (8.9)	1	0.95
33.92 – 34.23	50 (9.6)	1.09 (0.68 – 1.73)	
34.23 – 34.73	46 (8.9)	0.95 (0.60 – 1.52)	
34.73 – 36.79	45 (8.7)	0.91 (0.57 – 1.45)	
>36.79	51 (9.8)	1.07 (0.68 – 1.68)	
**BHR to methacholine**			
<33.92	59 (12.5)	1	0.29
33.92 – 34.23	69 (14.4)	1.08 (0.73 – 1.60)	
34.23 – 34.73	64 (13.3)	0.95 (0.64 – 1.41)	
34.73 – 36.79	65 (13.8)	1.03 (0.70 – 1.54)	
>36.79	53 (11.1)	0.81 (0.54 – 1.21)	
**Allergen skin sensitisation**			
<33.92	162 (31.2)	1	0.68
33.92 – 34.23	157 (30.2)	0.98 (0.74 – 1.30)	
34.23 – 34.73	160 (30.8)	1.02 (0.77 – 1.35)	
34.73 – 36.79	158 (30.4)	0.97 (0.73 – 1.28)	
>36.79	151 (29.1)	0.94 (0.72 – 1.24)	
**High total IgE**			
<33.92	114 (23.3)	1	0.22
33.92 – 34.23	117 (23.4)	0.98 (0.72 – 1.33)	
34.23 – 34.73	105 (21.6)	0.90 (0.66 – 1.23)	
34.73 – 36.79	110 (22.4)	0.90 (0.65 – 1.21)	
>36.79	103 (20.7)	0.84 (0.62 – 1.15)	

NO_2 _nitrogen dioxide; OR odds ratio; CI confidence interval; COPD chronic obstructive pulmonary disease; BHR bronchial hyper-responsiveness; IgE immunoglobulin E.

* OR adjusted for sex, age group, Carstairs deprivation score and smoking status

### Effect of traffic pollution on lung function measurements

In cross-sectional analyses, those living within 150 m of a main road were seen to have a similar FEV_1 _to those living further away, and amongst those living within 150 m of a road, there was no trend of reduced FEV_1 _with increased proximity (Table 5). Similarly, there was no association between measured values of lung function and modelled quintiles of NO_2 _at home location.

**Table 5:** Effect of proximity and modelled NO_2 _on cross-sectional FEV_1 _and longitudinal change in FEV_1_

	**Mean (unadjusted)**	**SD**	β*	**95% CI**	**p-value^†^**
**Cross-sectional**					
***Distance ***(N = 2599)					
≤150 m	3202.6	898.7	5.73	-42.80 to 54.27	0.82
>150 m	3178.5	924.1	0		

*Bands of distance *(N = 580)					
<50 m	3143.4	925.2	38.30	-63.60 to 140.21	0.47
50 – 100 m	3230.8	886.0	-10.65	-104.77 to 83.48	
100 – 150 m	3220.1	892.9	0		

***Quintile of NO*_2 _**(N = 2599)					
<33.92	3218.0	905.5	0		0.35
33.92 – 34.23	3163.6	940.1	-20.20	-85.00 to 44.59	
34.23 – 34.73	3147.0	927.5	-4.72	-69.41 to 59.96	
34.73 – 36.79	3213.5	937.9	9.79	-54.69 to 74.27	
>36.79	3177.2	880.9	18.12	-45.01 to 81.25	

**Longitudinal^‡^**	**Mean change (unadjusted)**	**SD**	β**	**95% CI**	**p-value^†^**
***Distance ***(N = 1329)					
≤150 m	-30.6	340.0	10.03	-33.98 to 54.04	0.65
>150 m	-36.5	334.0	0		

*Bands of distance *(N = 295)					
<50 m	-13.8	334.0	46.08	-53.31 to 145.46	0.35
50 – 100 m	-19.2	361.6	47.25	-47.43 to 141.93	
100 – 150 m	-59.1	320.7	0		

***Quintiles of NO*_2 _**(N = 1329)					
<33.92	-15.3	333.9	0		0.59
33.92 – 34.23	-24.5	334.3	-18.87	-76.85 to 39.11	
34.23 – 34.73	-41.8	329.1	-27.62	-85.68 to 30.45	
34.73 – 36.79	-57.6	327.1	-37.52	-96.16 to 21.12	
>36.79	-39.0	352.4	-21.58	-79.24 to 36.08	

SD standard deviation; β adjusted regression coefficient; CI confidence interval; NO_2 _nitrogen dioxide.

* β coefficient for cross-sectional FEV_1 _adjusted for sex, age, age squared, height, age-height interaction, smoking status, cigarette pack-years and Carstairs deprivation score, as recorded at baseline

** β coefficient for longitudinal change in FEV_1 _residuals adjusted for smoking status and cigarette pack years in 2000 and Carstairs deprivation score as recorded at baseline

† p-value for trend across quintiles of modelled NO_2 _and across bands of distance effects

‡ Change in lung function between 1991 and 2000

In longitudinal analyses of lung function over the 9 years of follow-up, decline in FEV_1 _was similar for those living within 150 m from the roadside and those living further away and showed no trend with proximity amongst those living within 150 m (Table 5). Similarly, there was no significant association between modelled NO_2 _and change in FEV_1 _(Table 5).

### Further analyses

Further control for other potential confounders, or occupation-based social class as an alternative to Carstairs deprivation score, did not materially alter any of the results, and restriction of cross-sectional and longitudinal analyses to the sub-group of long-term residents made little difference to the estimates. When total IgE was analysed as a continuous variable rather than a binary variable, no significant associations were seen with distance (adjusted β = -0.04, 95% CI -0.11 to 0.03 for <= 150 m relative to >150 m, p = 0.25) or modelled NO_2 _(p for trend = 0.69) (Table 6).

**Table 6:** Effect of proximity and modelled NO_2 _on Total IgE

**Exposure variable**	**N**	**Median Total IgE kU/l (IQR)**	Adjusted regression coefficient* (95% CI)	**P-value**
***Distance***				
<= 150 m	553	27 (8–80)	-0.04 (-0.11, 0.03)	0.25
>150	1914	26 (9–87.9)	0	
***Bands of distance***				
<50 m	153	23 (7–85)	0.05 (-0.11, 0.21)	0.61
50–100 m	219	30 (10–84)	0.07 (-0.07, 0.21)	P_trend _= 0.49
>100 m	181	27 (6–69.4)	0	
***Quintiles of NO_2 _(μg/m^3^)***				
<33.92	489	26.8 (9–88)	0	0.92
33.92–34.23	501	24 (9–91.5)	-0.04 (-0.13, 0.06)	P_trend _= 0.69
34.23–34.73	487	22 (9.8–80.8)	-0.01 (-0.11, 0.08)	
34.73–36.79	492	29.7 (9.7–85.1)	-0.02 (-0.11, 0.08)	
>36.79	498	26 (9–84)	-0.03 (-0.12, 0.06)	

IQR Interquartile range

* β coefficient from linear regression analysis of log transformed IgE

## Discussion

In this population-based study of Nottingham adults, we found no evidence that living close to a main road or in an area of increased traffic-related pollution was associated with an increased risk of asthma or COPD. This was true for both self-reported markers such as disease symptoms and diagnosis, and objective markers: BHR and lung function. Furthermore, in longitudinal analyses, there was no evidence that increased traffic exposure was associated with decline in lung function. We found some suggestion of an adverse effect of home proximity on allergy with a significant exposure-response across the first 150 m from roadside for allergic sensitisation, but not for other markers such as hayfever, eczema or total IgE.

The response rate, both to the original cross-sectional survey and in the 9 year follow-up of these subjects, was only approximately 50%, which raises the possibility of response bias. In the 2000 follow-up study, the characteristics of those who participated were generally similar to the original 1991 sample, and in particular, participation rates did not differ according to proximity to a main road. Whilst the factors associated with participation in the original 1991 survey are not fully known, proximity to a main road is not likely to be one of them since respondents and interviewers were unaware of the current hypothesis of investigation. We did find evidence that our study participants were slightly older than Gedling residents in the 1991 census[20], but proximity was not associated with age in our dataset (r = -0.03); socio-economic status was comparable to the census population and again was not related to proximity in our dataset (r = -0.03). Therefore, whilst we cannot completely rule out the possibility of response bias, it is unlikely to have had a major impact on our study results.

A strength of this study is that our exposure variables were computed using GIS techniques from the participant's exact address rather than postcode used in many previous studies. We estimated that by using the postcode rather than the exact address coordinates in the computation of the binary and the 50 m band distance variables, exposure status would have misclassified 5% and 33% of respondents respectively. In addition to the commonly used marker of exposure, residential distance from major roads, we used a more sophisticated marker of exposure based on modelled traffic-related NO_2 _concentrations. As this incorporated factors such as traffic patterns on the roads and meteorological influences, it is likely to be a more accurate marker of traffic pollution exposure. Whilst we have endeavoured to minimise misclassification by our choice of exposure variables, they still do not allow for exposure away from home and the possibility that they are insufficiently accurate to detect any true adverse effects that exist can not be ruled out. We also addressed the issue of our exposure variables being based on current (1991) home location only, which for those who had moved house may not be the relevant exposure. However our subjects had lived in their home for an average of 10 years and estimates were seen to remain similar when we restricted analyses to the subgroup of long term residents only, suggesting we had not missed any effects because of this issue.

Our findings for asthma fit with a number of previous studies of adults that also found no adverse effect of living in close proximity to a major road on self-reported symptoms [4-9]. One study that did report an adverse effect looked at US male veterans and showed that those living within 50 m of major roads had a increased risk of persistent wheeze, with a significant odds ratio of 1.7 for heavily trafficked roads (> = 10,000 vehicles a day), but a smaller effect (OR = 1.3) which reached borderline significance when all major roads were considered[1]. Two other recent studies have shown increased risks of wheeze of borderline significance in relation to increased residential proximity to surfaced roads in Ethiopia[2] and living within 20 m of a main street in Switzerland[3]. The Ethiopian study differed from most other studies in that it was conducted in a developing country where background pollution was thought to be very low, and it may be that the likelihood of detecting any real effects of home proximity to the roadside are greater in such settings. In our study, the sampling method and geographical area chosen are likely to have provided a sample broadly representative of the general population, but the fact that the Gedling district is primarily urban means that the majority of the sample live in areas with relatively high background concentrations of pollutants. Insufficient contrast in exposure may therefore explain why we were unable to detect any adverse effects of our localised traffic pollution markers in this study population. Comparison with a recent study in Rome that also modelled NO_2 _revealed less variation in our values (IQR 34.01 to 35.94 μg/m^3^) than those that experienced in Rome (IQR 37.3 to 50.3 μg/m^3^), although this study also found no positive associations with asthma either[7]. It is also possible that in some settings, effects of distance on asthma are evident across a wider range of distances than considered here, as suggested by Gauderman *et al *who reported a dose-response effect across the entire range of distance to the nearest freeway amongst children living in southern California [21].

A number of cross-sectional studies of lung function have, like ours, found no adverse effect of exposure to traffic on FEV_1 _[22-24]. However in a large study of US adults, Kan *et al *did find a negative association between traffic density at the residential location and FEV_1_, although in women only[25], and a similar finding was reported in a study of German woman in relation to living within 100 m of a major road[11]. In our study, no differential effects by gender were observed. As with asthma, adverse effects on lung function have been reported in Californian children in relation to much larger cut-points in distance (500 m bands), although again this was for freeways only [26]. Longitudinal studies of decline in FEV_1 _are more scarce, but in contrast to our finding of no effect, significant effects have been reported in relation to traffic-related pollution in Japanese women[9] and Swiss adults[27]. The latter used modelled PM_10 _concentrations, an exposure we were unable to analyse in our study as insufficient data were available for model validation. We also looked at spirometry-defined COPD using the same definition as that used previously by Schikowski *et al *who unlike us reported a significantly increased odds ratio of 1.79 in relation to living within 100 m of a busy roads[11]. Studies that looked at symptoms of COPD such as chronic cough and dyspnoea have generally found no significant associations[5,9].

Exposure to traffic pollution could plausibly increase the risk of sensitisation to allergens as traffic-related pollutants have been shown to enhance immunological responses to allergens[28]. Our finding of weak evidence of an effect on allergic sensitisation shows some consistency with that of Wyler and colleagues who reported an increased risk of skin sensitisation to pollen in relation to level of traffic at the home location[6]. However, they found no such effect on sensitisation to indoor allergens or hay fever[6]. Allergic sensitisation in adults was also investigated by Heinrich *et al *using specific IgE to common allergens and no relation to living near busy roads was seen[8]. We found no significant effect on hay fever or eczema in adults, which with one exception[7], is in agreement with others[2,5,6,8]. The lack of consistency of findings across different markers of allergy suggests caution is needed when interpreting one-off findings of adverse effects on allergic outcomes.

## Conclusion

In conclusion, we found no evidence to suggest that home proximity to major roads is a major determinant of the risk of asthma, COPD or allergic disease, or progression of obstructive lung disease in adults. However, because of relatively high levels of background pollution in our study area and possible misclassification of exposure, we cannot completely rule out an adverse effect, and further study is needed which incorporate life-time exposure to pollution in populations with wide variation in exposure.

## Competing interests

The authors declare that they have no competing interests.

## Authors' contributions

MP carried out all the statistical analyses, including data manipulation, interpreted the results and drafted the manuscript for publication. SL and TM supervised the data analysis, helped interpret the results and critically revised the manuscript. DW carried out the pollution modelling and creation of the NO_2 _exposure variable, helped interpret the results and critically revised the manuscript. JB was involved in the conception and design and in obtaining funding for the study. He also helped interpret the results and critically revise the manuscript. AV designed and obtained funding for the study, provided overall supervision for the study, including the data analysis and interpretation of results, and critically revised the manuscript for publication. All authors give final approval of the version of the manuscript submitted.

## Pre-publication history

The pre-publication history for this paper can be accessed here:



## References

[B1] Garshick E, Laden F, Hart JE, Caron A (2003). Residence near a major road and respiratory symptoms in U.S. Veterans. Epidemiology.

[B2] Venn A, Yemaneberhan H, Lewis S, Parry E, Britton J (2005). Proximity of the home to roads and the risk of wheeze in an Ethiopian population. Occup Environ Med.

[B3] Bayer-Oglesby L, Schindler C, Hazenkamp-von Arx M, Braun-Fahrlander C, Keidel D, Rapp R, Kunzli N, Braendli O, Burdet L, Liu S, Leuenberger P, Ackermann-Liebrich U, the SAPALDIA team (2006). Living near main streets and respiratory symptoms in adults. Am J Epidemiol.

[B4] Nitta H, Sato T, Nakai S, Maeda K, Aoki S, Ono M (1979). Respiratory health associated with exposure to automobile exhaust. I. Results of cross-sectional studies in 1982, and 1983. Arch Environ Health.

[B5] Oosterlee A, Drijver M, Lebret E, Brunekreef B (1996). Chronic respiratory symptoms in children and adults living along streets with high traffic density. Occup Environ Med.

[B6] Wyler C, Braun-Fahrlander C, Kunzli N, Schindler C, Ackermann-Liebrich U, Perruchoud AP, Leuenberger P, Wuthrich B (2000). Exposure to motor vehicle traffic and allergic sensitization. The Swiss Study on Air Pollution and Lung Diseases in Adults (SAPALDIA) Team. Epidemiology.

[B7] Cesaroni G, Badaloni C, Porta D, Forastiere F, Perucci CA (2008). Comparison between several indices of exposure to traffic-related air pollution and their respiratory health impact in adults. Occup Environ Med.

[B8] Heinrich J, Topp R, Gehring U, Thefeld T (2005). Traffic at residential address, respiratory health and atopy in adults: the National German Health Survey 1998. Environ Res.

[B9] Sekine K, Shima M, Nitta Y, Adachi M (2004). Long term effects of exposure to automobile exhaust on the pulmonary function of female adults in Tokyo, Japan. Occup Environ Med.

[B10] Gotschi T, Heinrich J, Sunyer J, Kunzli N (2008). Long-term effects of ambient air pollution on lung function: A review. Epidemiology.

[B11] Schikowski T, Sugiri D, Ranft U, Gehring U, Heinrich J, Wichmann HE, Kramer U (2005). Long-term air pollution exposure and living close to busy roads are associated with COPD in women. Respir Res.

[B12] Department of Transport, Scottish Office Industry Department, Office TW, Department of the Environment for Northern Ireland (1994). Environmental assessment (section 3). Design manual for roads and bridges.

[B13] Britton J, Pavord I, Richards K, Wisniewski A, Know A, Lewis S, Tattersfield A, Weiss S (1994). Dietary magnesium, lung function, wheezing, and airway hyperreactivity in a random adult population sample. Lancet.

[B14] Yan K, Salome C, Woolcock AJ (1983). Rapid method for measurement of bronchial responsiveness. Thorax.

[B15] McKeever TM, Scrivener S, Broadfield E, Jones Z, Britton J, Lewis SA (2002). Prospective study of diet and decline in lung function in a general population. Am J Respir Crit Care Med.

[B16] Ellis K, McHugh C, Carruthers D, Stidworthy A (2001). Comparison of ADMS-Roads, CALINE4 and UK DMRB Model Predictions for Roads. CERC Documentation.

[B17] Pauwels RA, Buist AS, Calverley PM, Jenkins CR, Hurd SS (2001). GOLD Scientific Committee: Global strategy for the diagnosis, management, and prevention of chronic obstructive pulmonary disease. NHLBI/WHO Global Initiative for Chronic Obstructive Lung Disease (GOLD) Workshop summary. Am J Respir Crit Care Med.

[B18] Carstairs V, Morris R (1989). Deprivation, mortality and resource allocation. Community Medicine.

[B19] Carey IM, Strachan DP, Cook DG (1998). Effects of changes in fresh fruit consumption on ventilatory function in healthy British adults. Am J Respir Crit Care Med.

[B20] Office of Population Censuses and Surveys, 1991 Census Small Area and Local Base Statistics (England and Wales) ESRC/JISC Census Programme, Census Dissemination Unit, Mimas (University of Manchester).

[B21] Gauderman WJ, Avol E, Lurmann F, Kuenzli N, Gilliland F, Peters J, McConnell R (2005). Childhood asthma and exposure to traffic and nitrogen dioxide. Epidemiology.

[B22] Maeda K, Nitta H, Nakai S (1991). Exposure to nitrogen oxides and other air pollutants from automobiles. Public Health Rev.

[B23] Nakai S, Nitta H, Maeda K (1987). Respiratory health associated with exposure to automobile exhaust. III. Results of a cross-sectional study in and repeated pulmonary function tests from 1987 to 1990. Arch Environ Health.

[B24] Franco Suglia S, Gryparis A, Schwartz J, Wright RJ (2008). Association between traffic-related black carbon exposure and lung function among urban women. Environ Health Perspect.

[B25] Kan H, Heiss G, Rose KM, Whitsel E, Lurmann F, London SJ (2007). Traffic exposure and lung function in adults: the Atherosclerosis Risk in Communities study. Thorax.

[B26] Gauderman WJ, Vora H, McConnell R, Berhane K, Gilliland F, Thomas D, Lurmann F, Avol E, Kunzli N, Jerrett M, Peters J (2007). Effect of exposure to traffic on lung development from 10 to 18 years of age: a cohort study. Lancet.

[B27] Downs SH, Schindler C, Liu LJ, Keidel D, Bayer-Oglesby L, Brutsche MH, Gerbase MW, Keller R, Künzli N, Leuenberger P, Probst-Hensch NM, Tschopp JM, Zellweger JP, Rochat T, Schwartz J, Ackermann-Liebrich U, SAPALDIA Team (2007). Reduced exposure to PM10 and attenuated age-related decline in lung function. N Engl J Med.

[B28] Bartra J, Mullol J, del Cuvillo A, Dávila I, Ferrer M, Jáuregui I, Montoro J, Sastre J, Valero A (2007). Air pollution and allergens. J Investig Allergol Clin Immunol.

